# EXPERIENCES AND SUPPORTIVE CARE NEEDS OF LATINX MILLENNIAL CAREGIVERS

**DOI:** 10.1093/geroni/igad104.3101

**Published:** 2023-12-21

**Authors:** Anna Welling, Megan Thomas Hebdon, Galilea Dupree, Sharon Horner, Michael Thomas, Neil Peterson, Janice Hernandez, Heather Cuevas

**Affiliations:** The University of Texas at Austin, Austin, Texas, United States; The University of Texas at Austin, Austin, Texas, United States; The University of Texas at Austin, Austin, Texas, United States; The University of Texas at Austin, Austin, Texas, United States; The University of Texas at Austin, Austin, Texas, United States; Brigham Young University, Provo, Utah, United States; The University of Texas at Austin, Austin, Texas, United States; The University of Texas at Austin, Austin, Texas, United States

## Abstract

Latinx millennial caregivers are the largest ethnic minority subgroup within the millennial caregiving cohort. Due to age and life stage, members of this understudied group must often manage the competing priorities of career, family, and caregiving. Layered with these challenges are the experiences of being part of a minority group in the United States, with systemic barriers impacting access to healthcare, insurance, a safe built environment, and educational opportunities. The purpose of this qualitative descriptive study was to explore the experiences and needs of Latinx millennial caregivers. Participants were recruited locally and nationally using recruitment flyers and social media advertising. Data were collected from 29 participants using five focus group interviews and one individual interview. Data analysis using interpretive thematic analysis and inductive coding occurred. Twenty female and nine male Latinx millennial caregivers participated in the focus groups. Two meta-themes threaded throughout the analysis included the Latinx experience (immigrant background, Latinx culture, and navigating barriers as a minoritized individual) and being a super-caregiver. Main themes were identified: family well-being, occupational and financial well-being, social support dynamics, challenges and rewards of family caregiving, and coping strategies. Family and community were emphasized in all main themes. In this study, the importance of family and community relationships motivated caregiving and were areas where support was needed for Latinx millennial caregivers. Interventions and clinical support for these caregivers is needed that addresses their cultural background, the value of family and community, and the barriers they experience to accessing care and support.

